# Recent advancement of glucosamine and N-acetyl glucosamine production using microorganisms: A review

**DOI:** 10.1093/jimb/kuaf014

**Published:** 2025-05-27

**Authors:** Anica Tasnim Protity, Shengde Zhou

**Affiliations:** Department of Biochemistry and Microbiology, North South University, Dhaka, Bangladesh; Department of Biological Sciences, Northern Illinois University, Dekalb, IL 60115, USA; Department of Biological Sciences, Northern Illinois University, Dekalb, IL 60115, USA

**Keywords:** Osteoarthritis, Glucosamine (GlcN), N-acetyl glucosamine (GlcNAc), Metabolic engineering, Fermentation

## Abstract

Glucosamine (GlcN) and GlcN-based supplements, e.g. glucosamine hydrochloride, glucosamine sulfate, and N-acetyl glucosamine (GlcNAc), provide symptomatic relief to osteoarthritis patients and have been used as one of the most popular nutraceuticals. To meet the increasing demands, scientists have explored cost-effective methods for GlcN and GlcNAc production using low-cost raw materials such as seafood waste. However, the commercially available GlcN and GlcNAc production methods are environmentally harmful because of the use of toxic reagents. Moreover, the raw material used might be unsafe for consumers with shrimp allergies. On the other hand, bio-based GlcN production is gaining popularity because of its eco-friendly production approach and optimum reaction conditions. In this mini-review, we will discuss the recent developments to produce GlcN and GlcNAc through (1) the chemical and enzyme-mediated approaches of crude chitin hydrolysis, primarily obtained from shrimp and crabs; (2) the whole cell-based systems for fungal derived chitin bio-transformation and fungal fermentation; and (3) the metabolic engineering and the adaptive evolution based microbial biocatalyst for a balanced cell growth and optimal production of GlcN and GlcNAc.

**One-Sentence Summary:** This article summarizes the mechanism of glucosamine and N-acetyl glucosamine production using bacteria, fungi, and chemical processes.

## Graphical Abstract

**Graphical Abstract:**
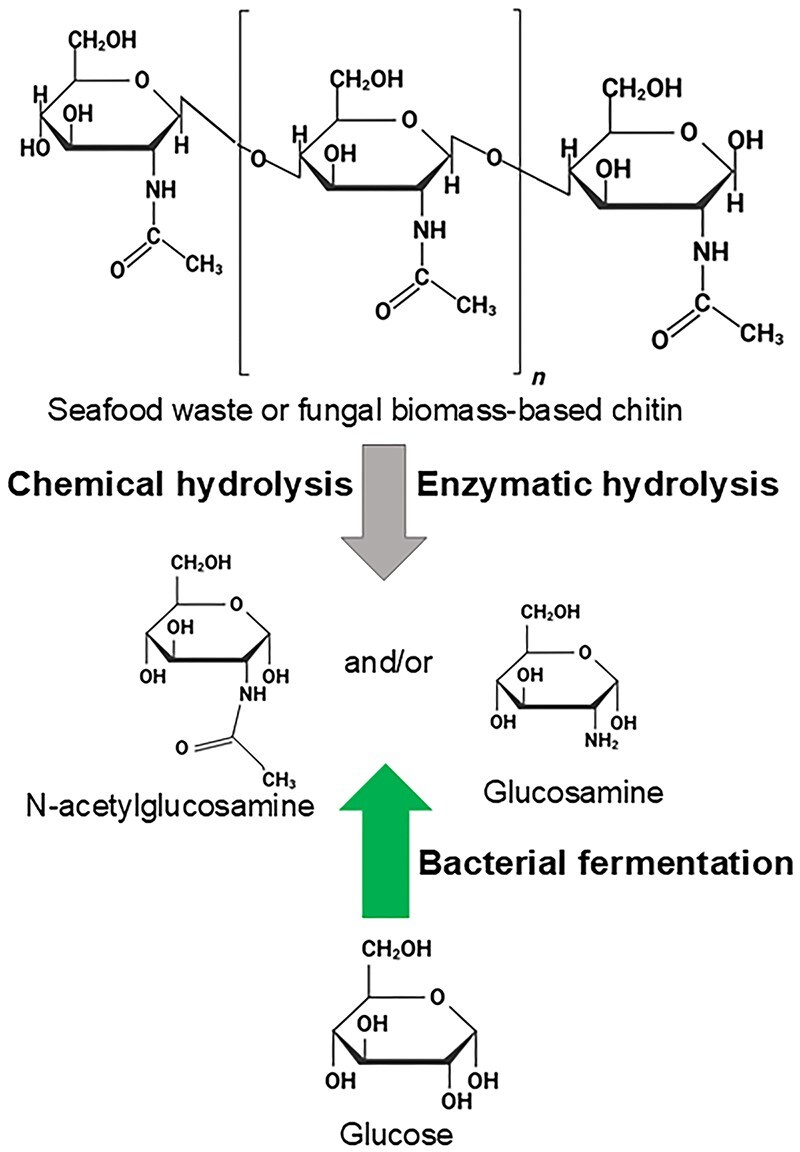

## Introduction

### Glucosamine and N-acetyl Glucosamine

**Fig. 1.**
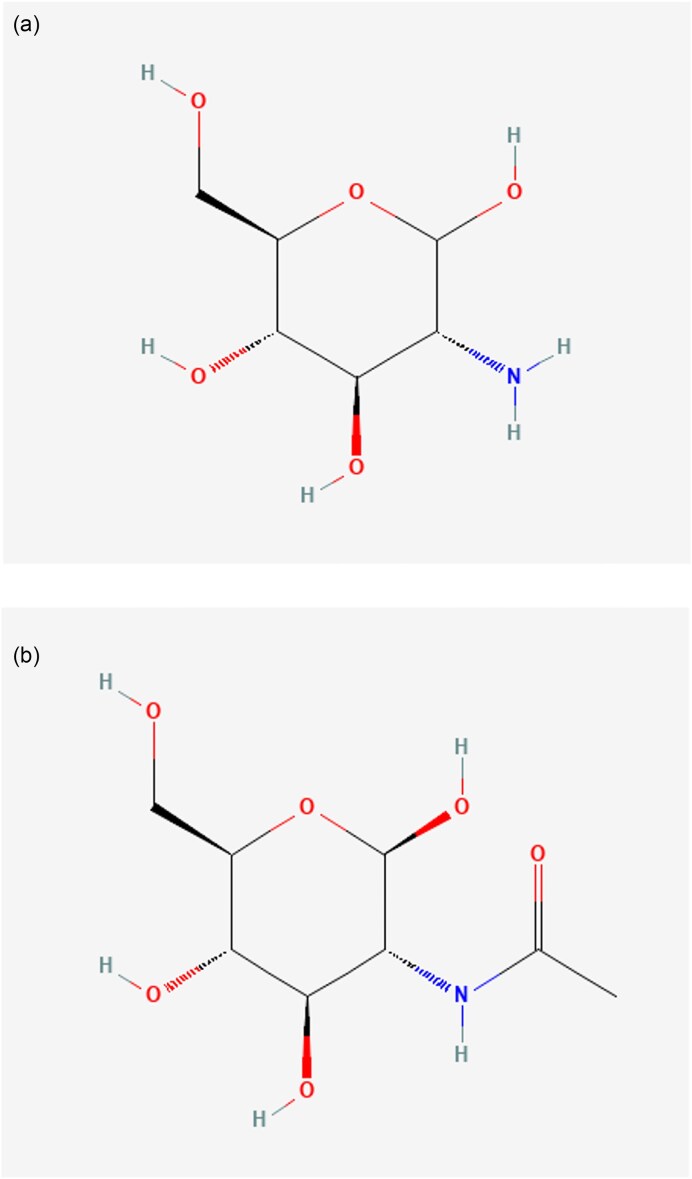
(a) Chemical structure of Glucosamine (GlcN). (b) Chemical structure of N-acetyl glucosamine (GlcNAc).

Glucosamine (GlcN) is an amino sugar widely distributed in nature. This molecule was discovered in 1876 from chitin by Georg Ledderhose, who named it glycosamine (Ledderhose, 1876). Later in 1902, it was synthesized and purified by Hermann Leuchs, a student of Emil Fischer. GlcN is chemically formed by replacing one hydroxy group of a glucose molecule with an amino group. Besides being a chitin monomer, GlcN is a precursor for synthesizing glycosaminoglycans (GAGs), namely, hyaluronic acid, heparan sulfate, and keratan sulfate. All these components are found in the synovial fluid in the joint cavities, cartilage, and other connective tissues (Jerosch, 2011; Malik et al., 2013). N-acetyl glucosamine (GlcNAc), GlcN hydrochloride, and GlcN sulfate are three major derivatives of GlcN-based nutraceuticals available in oral formulation. These supplements provide regeneration of damaged cartilage, reduction of joint inflammation, and prevention of bone-joint structural change in osteoarthritis (OA) patients (Deal & Moskowitz, 1999; Deng et al., 2005; Liu et al., 2013; Noack et al., 1994). GlcNAc is an important amino sugar with numerous industrial and medical applications, including its use in the food and pharmaceutical industries for its anti-inflammatory and immunomodulatory properties (Chen et al., 2010). Being a building block of hyaluronic acid, GlcNAc has also been used for dermatological benefits in the skincare industry, such as wound healing, skin hydration, and antiwrinkle treatment (Chen et al., 2010). The chemical structures of GlcN and GlcNAc are illustrated in Fig. 1a and b, respectively.

According to a study conducted in 2007, GlcN was the second most used nonvitamin, nonmineral complementary, and an alternate medicine (CAM) for adults in the US (Barnes et al., 2008). More than five million people in the US are taking GlcN supplements (Kennedy, 2005). In Australia, GlcN-based supplements are suggested by 94.7% of Australian community pharmacists (Sibbritt et al., 2012). The global GlcN market size was estimated by Coherent Market Insights (2020) to be $763.7 million in 2020 and about $1 billion by 2027. According to Grand View Research, US is the largest consumer of GlcN, with an estimated market size of $319 million in 2020 (Nutraceutical Market Size Worth $722.49 Billion by 2027, 2025). Although GlcN-based supplements are becoming more popular in Asia and Europe, the US is still the largest consumer in the market, with China serving as the primary manufacturer (Liu et al., 2013).

### Health Benefits of GlcN and GlcNAc Based Nutraceuticals

The use of GlcN as an oral supplement started about 40 years ago, with the first report of significant symptomatic relief among the patients receiving GlcN oral pills (D'Ambrosio et al., 1981). Some groups of scientists found out that long-term use of GlcN and its derivatives alleviated arthritis pain (McAlindon et al., 2000; Pavelk´a et al., 2002; Reginster et al., 2001; Richy et al., 2003; Zhu et al., 2018). According to the Glucosamine/Chondroitin Arthritis Intervention Trial (GAIT), 79% of individuals having moderate to severe joint pain had a significant pain reduction upon oral administration of GlcN and chondroitin sulfate (The NIH Glucosamine/Chondroitin Arthritis Intervention Trial (GAIT), 2008). GlcN-based nonvitamin, nonmineral supplements are commonly used for OA pain and are often taken with chondroitin (Jerosch, 2011). OA is a degenerative joint disease that occurs when the protective cartilage that cushions the ends of bones wears down over time, causing pain, stiffness, and loss of mobility. It is the most common form of arthritis, affecting over 32.5 million adults in the US (CDC, 2024). Currently, there is no definite medicine for OA. GlcN, present in healthy joint cartilage, might play a role in cartilage repair and maintenance (Noack et al., 1994; Sibbritt et al., 2012; Yuan et al., 2021). GlcN may promote the formation of GAGs, slowing down the breakdown of the extracellular matrix (Yamada et al., 2022). Studies have shown that taking GlcN supplements may help prevent the development or progression of OA by reducing inflammation, improving joint mobility, and supporting cartilage regeneration (Deal & Moskowitz, 1999; Ma et al., 2020; Zhang et al., 2021). As an anti-OA food supplement, GlcN, either administered alone or in combination regularly, lowers the inflammation in OA patients and is considered a safe CAM to use (Zhang et al., 2021; Moon et al., 2021).

Several studies proved the efficacy of GlcN supplements in alleviating OA pain compared to the NSAID treatments (Jerosch, 2011; Noack et al., 1994; Reginster et al., 2001; Towheed & Wells, 2005). These products have passed clinical trials and have been approved by the FDA as over-the-counter supplementary drugs for elderly individuals to prevent premature loss of cartilage, knee pain, and back pain (Deng et al., 2005; Liu et al., 2013; Malik et al., 2013). A study with healthy soccer players showed that GlcN administration significantly decreased collagen degradation by minimizing the level of collagen-degrading biomarkers in urine samples (Tsuruta et al., 2018). Based on its safety profile, all the studies have announced GlcN administration as safe (Chan et al., 2010; Hochberg et al., 2016; Reginster et al., 2001; Towheed & Wells, 2005).

## Production Routes of GlcN and GlcNAc

There are several methods for producing GlcN, including acid hydrolysis of crustacean shells, enzymatic degradation of chitin and chitosan, and biobased microbial fermentation. Acid hydrolysis involves using concentrated acids, such as hydrochloric acid or sulfuric acid, to break down chitin, a polymer found in the exoskeletons of shellfish, into GlcN (Benavente et al., 2015; Mojarrad et al., 2007). Enzyme-mediated hydrolysis involves multiple enzymes to break down colloidal chitin and chitosan molecules obtained from various sources (langostino shell, squid pen, and fungal cell wall), into GlcNAc and GlcN monomers, respectively (Donzelli et al., 2003; Sashiwa et al., 2003; Subramanyam & Rao, 1987). Biobased microbial production involves using microorganisms, mostly fungi or bacteria, to produce GlcN and GlcNAc through fermentation of various carbon sources (Chen et al., 2010; Deng et al., 2005; Zhang et al., 2020).

### Production of GlcN and GlcNAc By Acid Hydrolysis

**Fig. 2.**
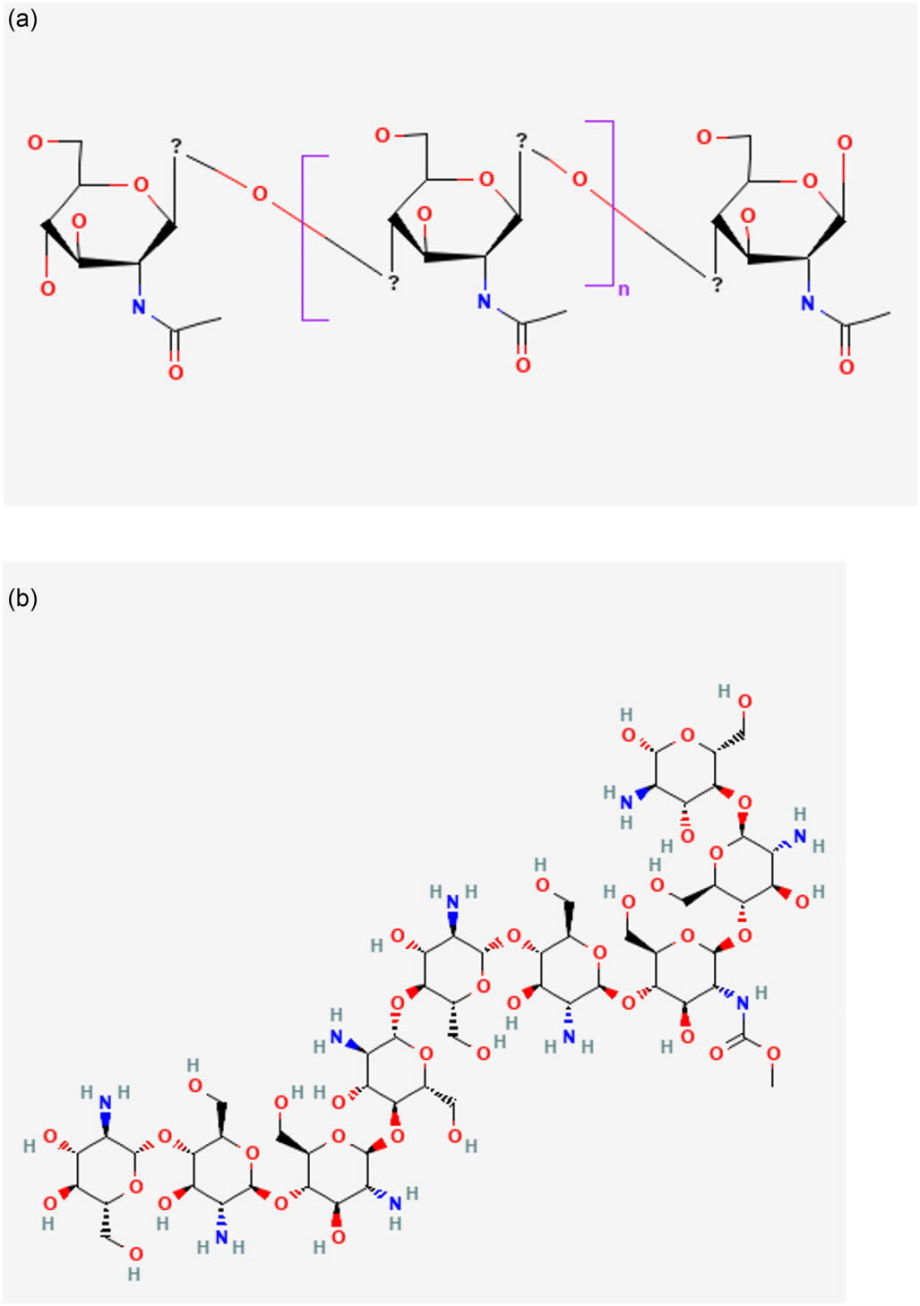
(a) Chemical structure of chitin. (b) Chemical structure of chitosan.

Conventionally, GlcN and GlcNAc are produced via acid hydrolysis of chitin and chitosan (Benavente et al., 2015; Ferrer et al., 1996; Liu et al., 2013; Mojarrad et al., 2007; Sitanggang et al., 2012). Chitin is the second largest naturally occurring biopolymer after cellulose, found in the exoskeletons of crustaceans, insects, and some fungi (Yadav et al., 2019). Chitosan is a derivative of chitin that is produced by deacetylation. The cleavage of the glycosidic bond and the deacetylation of chitin yield a D-GlcN monomer. The structures of chitin and chitosan are illustrated in Fig. 2a and b, respectively.

Both chitin and chitosan can be extracted for GlcN production. This extraction procedure involves several steps. First, chitin or chitosan is purified and demineralized to remove impurities. The purified material is then deproteinized and depigmented before being treated with acid to release GlcN. The resulting product is then further purified to remove the remaining impurities (Benavente et al., 2015; Mojarrad et al., 2007). Large quantities of waste are produced during the industrial processing of crustaceans for food production, of which 50%–60% of the dry weight is made up of seafood shells. Approximately 6–8 million tons of crab shells are produced worldwide each year, of which 15%–40% of the weight is chitin (Yan & Chen, 2015). The primary commercial source of chitin is shrimp and crab shells found in solid seafood waste (Shahidi et al., 1999). However, another paper proposed a general protocol for obtaining D-GlcN from shrimp shells, cicada sloughs, and cockroaches (Bertuzzi et al., 2018), although the yield is lower, having 13.70%, 20.46%, and 3.28% GlcN from shrimp shells, cicada sloughs, and cockroaches, respectively. Chitin extracted from seafood waste can be hydrolyzed at a high temperature using a powerful acid and alkali to produce GlcN and its derivatives (Deng et al., 2005; Liu et al., 2013; Sitanggang et al., 2012). However, at neutral pH, amino sugars with a free amino group are unstable in aqueous solution. GlcN has a free amino (-NH₂) group at the second carbon (C2). On the contrary, GlcNAc does not have a free amino group, because the amino (-NH₂) group is acetylated (-NHCOCH₃). Therefore, GlcN can spontaneously rearrange and dimerize to create compounds like fructosazine, D-arabinose, 5-(hydroxymethyl)-2-furaldehyde, 2,5-bis (tetrahydroxybutyl) pyrazine, etc (Deng et al., 2005). Hence, it is precipitated in salt, such as GlcN sulfate sodium chloride.

**Fig. 3.**
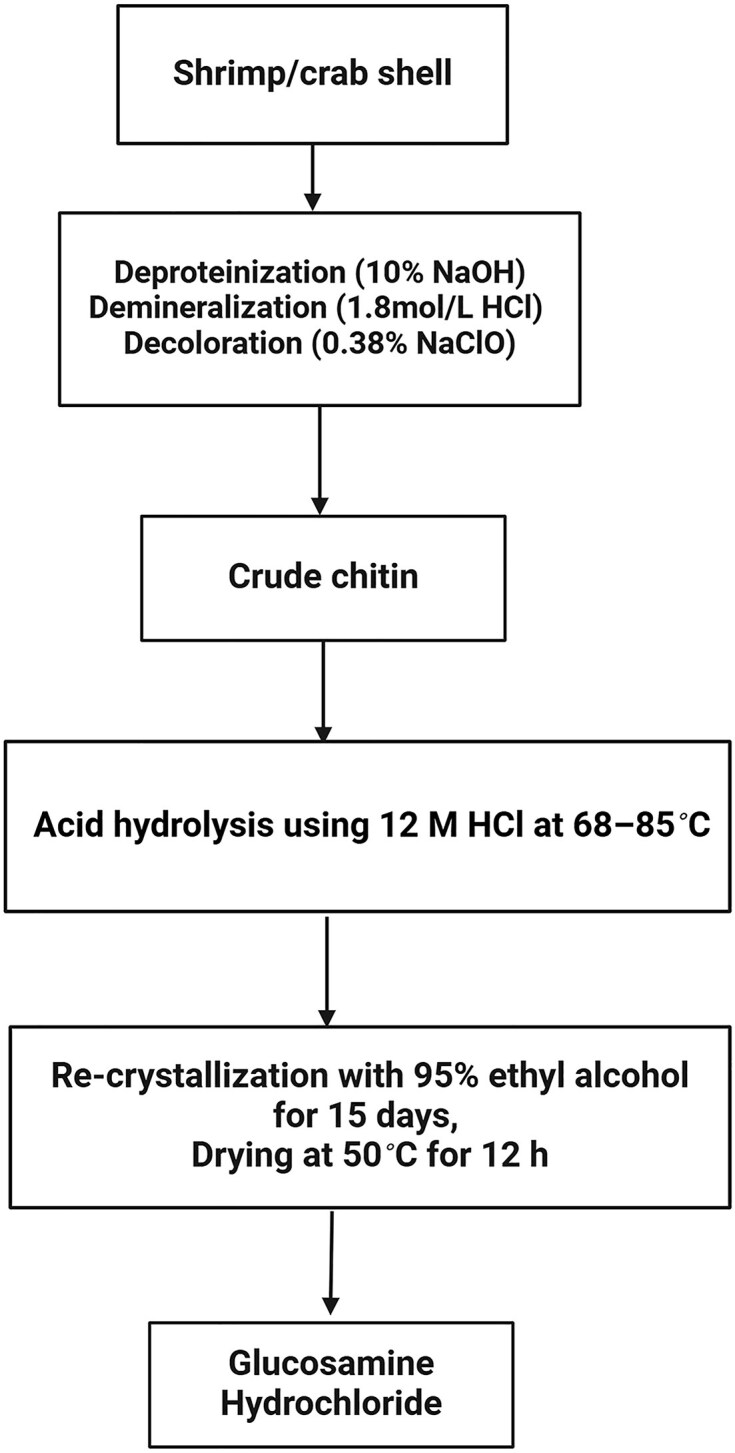
Schematic production of GlcN-Cl using acid hydrolysis method.

According to Benavente et al., GlcN was obtained in the form of GlcN hydrochloride by several steps, including deproteination of the crustacean exoskeleton (head, legs, shell, and tail) using 10% NaOH, demineralization of the crude chitin using 1.8 M HCl for 12 hr, depigmentation using bleach, drying, filtration, and acid hydrolysis of the filtrated product using 12 M HCl at 68°C–85°C. The yield (GlcN hydrochloride/chitin) was about 58% for a 1:20 solid/liquid ratio (Benavente et al., 2015). Another study improved GlcN yield by 96%–98% using different optimization parameters, such as 37% HCl, a 9:1 ratio of acid/solid, and 4 hr of operation time (Mojarrad et al., 2007). Besides, Zhang et al. showed that 80% GlcN could be produced from ball-milled chitin using a low quantity of sulfuric acid at 175°C for 1 hr and diethylene glycol diethyl ether as a co-solvent (Zhang & Yan, 2017). The generic flowchart for acidic hydrolysis of GlcN is illustrated in Fig. 3.

Like GlcN production, GlcNAc is industrially produced from acid hydrolysis of crude chitin, using HCl and carefully scrutinizing reaction temperature and acid concentration to prevent end-product degradation. The productivity was up to 6.42 g/L/hr of GlcNAc (Chen et al., 2010). However, high cost, low yield, and the production of acidic waste complicated the treatment process. An alternative process involves the acetylation of GlcN by dissolving chitin in concentrated HCl, boiling it in water for 3 hr, and performing an additional N-acetylation reaction (Chen et al., 2010). This chemical synthesis involves the reaction of GlcN with acetic anhydride to form GlcNAc (Kohn et al., 1962). Although efficient, this method, along with other acid hydrolysis methods, requires toxic and hazardous chemicals, which can pose a risk to workers and the environment. The steps involved in these processes include HCl, H_2_SO_4_, NaOH, and NaClO; all of which exhibit strong corrosive and toxic properties, such as severe burns, respiratory damage, irreversible blindness, even life-threatening complications after ingestion. Moreover, upon release into the environment, the aquatic ecosystem gets severely affected by the toxicity of these chemicals.

In conclusion, although the chemical GlcN and GlcNAc production processes can quickly generate a higher yield and higher purity product, these methods contain certain drawbacks, such as variable raw material supply throughout the year, indefinite size and composition of shellfish, the role of shellfish as a potential allergen to some consumers, complicated pretreatment of raw materials, multistep hydrolysis process, harsh chemical and high-temperature treatment conditions, and large industrial waste load (Deng et al., 2005; Liu et al., 2013; Sitanggang et al., 2012).

### Production of GlcN and GlcNAc by Enzymatic Hydrolysis

**Fig. 4.**
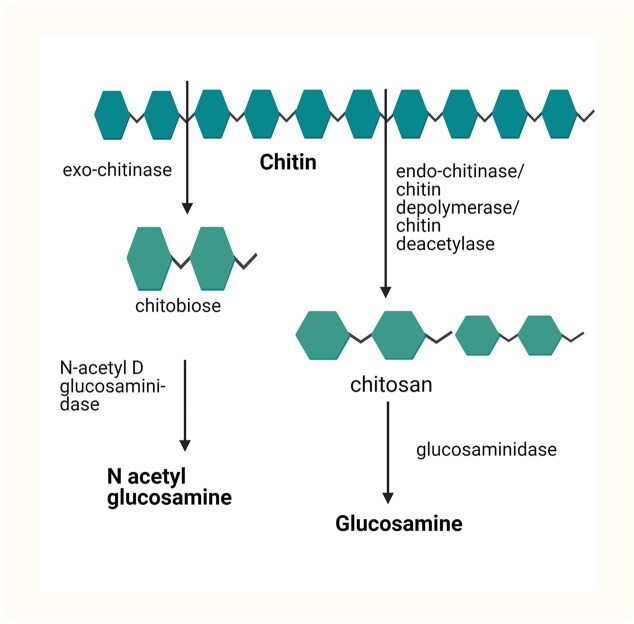
Enzyme-mediated hydrolysis of GlcN and GlcNAc (https://BioRender.com/v38p345).

Developing eco-friendly methods for producing GlcN and GlcNAc has been established for the last 20 years (Matsumoto et al., 2004; Sitanggang et al., 2010). These methods primarily involve using bacterial or fungal-derived enzymes to hydrolyze chitin and chitosan molecules. The enzymatic process is carried out in several steps, which involve the solubilization of chitin, the production of short oligomers, and the conversion of oligomers into GlcN or GlcNAc (Chen et al., 2010; Neeraja et al., 2010). Various studies have explored this enzymatic approach for producing GlcN and GlcNAc using different types of enzymes. As a polysaccharide, chitin is a linear chain of β-1,4-N-acetyl-D-GlcN monomers. Four enzymes are involved in the chitin hydrolysis process: endochitinases, exochitinases, chitobiases, and β-N-acetylhexosaminidases (Liu et al., 2013). Endochitinases randomly cleave the internal chain of chitin, producing low-molecular-weight oligomers of GlcNAc, such as chitotetraoses and chitotrioses, eventually leading to diacetyl chitobiose as the main product. On the other hand, exochitinases release diacetylchitobiose without producing GlcNAc or oligomers. β-N-acetylhexosaminidase acts on diacetylchitobiose. Diacetylchitobiose, chitotriose, and chitotetraose can all be split into GlcNAc monomers by β-N-acetylhexosaminidases (Liu et al., 2013). The enzyme hydrolysis method is illustrated in Fig. 4.

GlcNAc can be enzymatically hydrolyzed from squid pen chitin, a form of β-chitin extracted from the pen (gladius) of squid, using chitinase from *Aspergillus* sp., with a maximum yield of 65% (Setthakaset et al., 2008). According to Neeraja et al., the enzymatic hydrolysis of chitin using a chitinase produced by *Bacillus cereus* MTCC 6840 at pH 6.5 and a temperature of 50°C resulted in a 91% yield of GlcNAc after 24 hr (Neeraja et al., 2010). A recent study explored the optimal hydrolysis conditions of shrimp colloidal chitin using extracellular enzymes produced by a marine bacterium (PRO-III 115), achieving the highest GlcNAc yield of 2.65% using 10 mg/mL colloidal chitin, at a temperature of 60°C, a pH of 8.9, and 3.5% NaCl (Rivera-Sols et al., 2023). Another study developed an eco-friendly whole-cell biocatalytic method for converting GlcNAc to GlcN using recombinant *Bacillus subtilis* expressing diacetylchitobiose deacetylase enzyme from an archaeon called *Pyrococcus furiosus*. The gene encoding this enzyme, while overexpressed into *B. subtilis* showed superior performance, achieving a maximum GlcN titer of 35.3 g/L with an 86.8% molar conversion ratio under optimized conditions in a 3-L bioreactor (Zhu Jiang et al., 2018).

Enzyme-mediated chitin hydrolysis occurs under mild conditions, can be repeatedly used as an immobilized catalyst, and enables GlcN production from nonanimal sources, such as bacteria and fungi, which is beneficial for consumers with crustacean allergies and vegan diets. However, it has some significant setbacks compared to the acid hydrolysis method, such as incomplete bioconversion of the raw material, poor product yield, enzyme degradation, low enzyme activity, and a high enzyme cost (Chen et al., 2010; Deng et al., 2005; Liu et al., 2013). A cell-mediated production of GlcN and GlcNAc may be able to circumvent this issue.

### Production of GlcN and GlcNAc by Fungal Fermentation

Fungal cell wall comprises chitin and chitosan, which are polymers of GlcNAc and GlcN, respectively. Therefore, submerged (SF) and solid-state fermentation (SSF) of filamentous fungi can be used as potential resources to produce GlcN and GlcNAc. Examples of fungi in *Ascomycotina* and *Zygomycotina* groups that have been reported to produce GlcN include *Aspergillus* sp., *Rhizopus* sp., and *Mucor* sp. (Hsieh et al., 2007; Sitanggang et al., 2010; Zhang et al., 2012). Usually, the overall steps include fungal strain selection, inoculum preparation, fermentation process, separation of fungal biomass from the media, and hydrolysis of the biomass to yield GlcN. *Aspergillus* sp. has been reported to produce up to 7.48 g/L of GlcN, and up to 14.37 g/L of GlcN can be produced through SF and SSF using *Aspergillus* sp. BCRC31742 (Hsieh et al., 2007). The biomass of *Aspergillus oryzae* NCH-42 has a high potential for GlcN production, as its GlcN content can reach up to 0.31 gm per gram of biomass (Li et al., 2021). Another study reported an increase in the yield and productivity of GlcN using a low-cost solid-state culture of *A. sydowii* BCRC 31742 by optimizing fermentation conditions. The maximum yield was 48.7 mg of GlcN per gram of dry substrate (Peng & Wu, 2020). Additionally, *A. terreus*, a marine fungus, has been found to produce GlcNAc, which accounts for 92% of total fermentation products (Das et al., 2019). Matsumoto et al. produced GlcNAc from seafood waste using ß-N-acetylhexosaminidase from *Verticillium lecanii*, grown through SF and SSF (Matsumoto et al., 2004).

However, fungal fermentation has certain limitations, such as low product yield, the need for GlcN and GlcNAc extraction from fungal mycelia after fermentation, and varying chitin content between fungal species, making it less economically competitive compared to traditional acid hydrolysis of chitin and chitosan from crab and shrimp shells (Liu et al., 2013).

## GlcN and GlcNAc Synthesis in Bacteria

GlcNAc is a major peptidoglycan building block in the cell walls of Gram-positive and Gram-negative bacteria. Bacterial fermentation is a promising technique for producing GlcN and GlcNAc that could lower production costs while maintaining consistent quality and increased yield as compared to fungal fermentation and other biobased methods of GlcN and GlcNAc synthesis. Bacterial production of GlcN and GlcNAc involves the selection of microbial strains, genetic and metabolic engineering for maximized production, optimization of fermentation conditions, and downstream processing.

### Host and Strain Selection

Selecting an appropriate microbial host is crucial, as the strain should have a high growth rate, tolerate the fermentation conditions, and mediate an effective conversion of glucose to GlcN and GlcNAc. Several studies reported the use of recombinant *E. coli, B. subtilis*, and *Corynebacterium glutamicum* to produce GlcN and GlcNAc (Deng et al., 2019, 2005; Lu et al., 2022; Ma et al., 2021). *E. coli* is a well-characterized strain that is easy to manipulate genetically and has a high growth rate (Deng et al., 2005; Lu et al., 2022). More importantly, *E. coli* can directly secrete GlcN and GlcNAc into the fermentation media, making product purification much easier. Due to these traits, recent research has focused on developing *E. coli* strains to produce GlcN and GlcNAc (Chen et al., 2012; Ma et al., 2021; Wang et al., 2022).

### Metabolic Engineering of *E. coli* Strains for GlcN and GlcNAc Production

Metabolic engineering is used to modify existing cellular metabolic pathways for the overproduction of desired metabolites (Kumar & Prasad, 2011). Microorganisms have been a primary focus of metabolic engineering because they can produce many compounds of industrial interest. As the natural metabolic network of microbial cells is not optimized for practical applications, attempts to use wild-type organisms for industrial production were hindered by low yields and high costs. Metabolic engineering has been employed to overcome these issues (Kumar & Prasad, 2011).

**Fig. 5.**
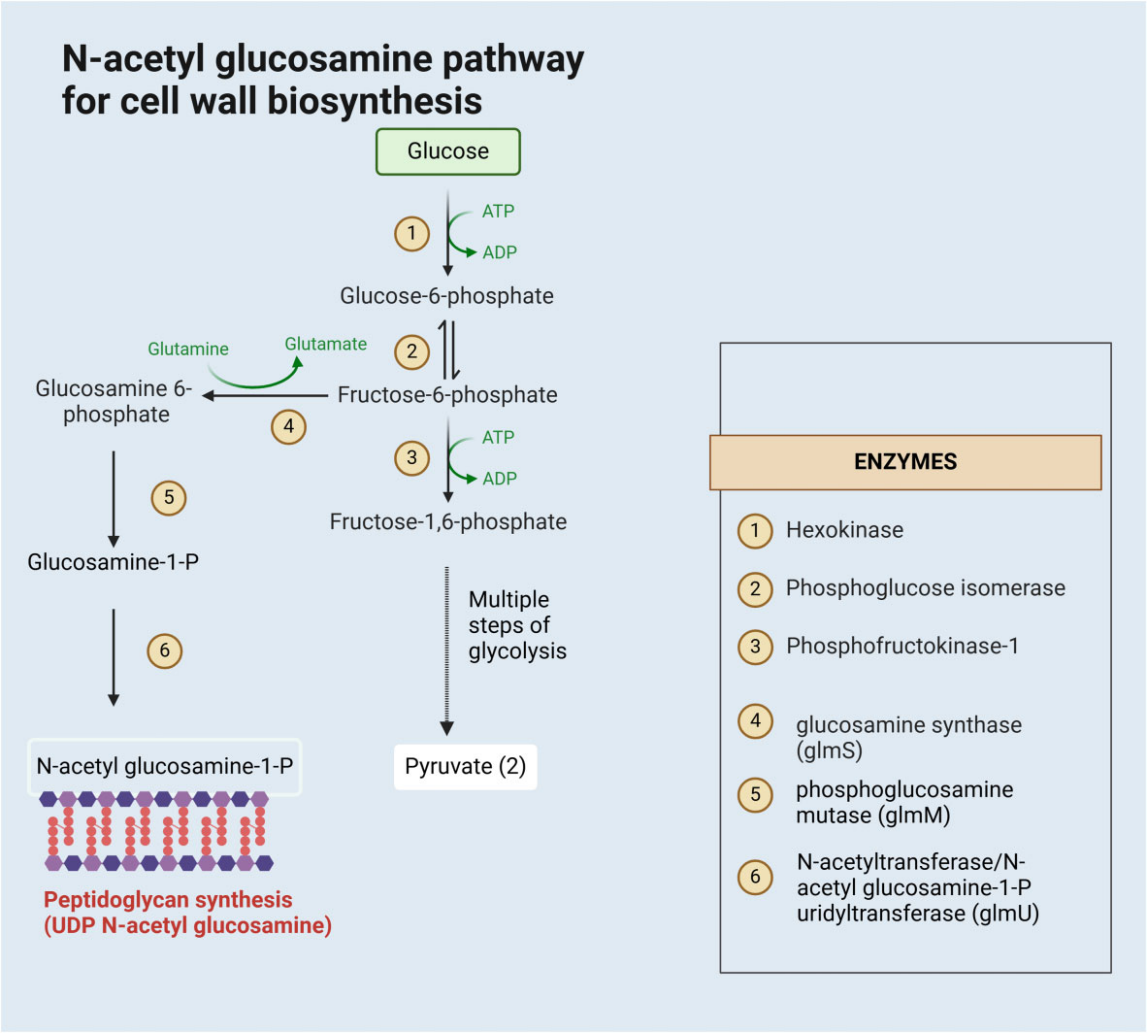
Intrinsic pathway of *E. coli* for GlcNAc production for cell wall synthesis (https://BioRender.com/u40k928).

*Escherichia coli* has an intrinsic biosynthetic pathway for GlcN and GlcNAc production. Illustrated in Fig. 5, this pathway has multiple steps. Firstly, fructose-6-phosphate is converted into GlcN-6-phosphate by an enzyme called GlcN synthase (L-Glutamine: D-fructose-6-phosphate amidotransferase), encoded by the *glmS* gene, using glutamine as an amine donor. GlcN-6-P is then converted to GlcN-1-P by phosphoglucosamine mutase (*glmM*). This GlcN-1-P is further converted into UDP-GlcNAc by N-acetyltransferase/GlcNAc-1-P uridyltransferase (*glmU*) enzyme. GlcN-6-P and GlcNAc-6-P can be dephosphorylated and secreted outside the cell using an unknown exporter (Liu et al., 2013).

As an essential gene, *glmS* expression is highly regulated to produce an optimal amount of GlcN for cell growth. Downregulation of *glmS* mRNA and protein occurs in the presence of amino sugars (Plumbridge et al., 1993). Co-transcription of *glmS* and *glmU* generates the *glmUS* transcript, which undergoes post-transcriptional modification by RNAse E to produce a highly unstable 1.9 kb monocistronic *glmS* transcript (Joanny et al., 2007). This *glmS* transcript expression is also post-transcriptionally regulated by two small RNAs, GlmY and GlmZ (Kalamorz et al., 2007; Urban et al., 2007). This highly regulated *glmS* expression is necessary to synthesize GlcNAc as a cell wall component.

RNA structure known as the glmS ribozyme is found in the 5' untranslated region (UTR) of the mRNA transcript of the *glmS* gene. At high concentration of GlcN-6-P levels inside the cell, the glmS ribozyme is activated, and the mRNA transcript is cleaved. When the GlcN-6-P level is scarce, the gene is translated into GlcN synthase, and GlcN6P is produced (Winkler et al., 2004).

It is a disadvantage for the production of GlcN as a sole fermentation product. To overcome the regulation, an additional copy of the *glmS* gene is usually inserted into a recombinant bacterial host strain for GlcN overproduction (Deng et al., 2005; Liu et al., 2013).

However, *glmS* overexpression would increase the intracellular GlcN-6-phosphate concentration, which has intrinsic toxicity, leading to inhibition of cell growth and a decreased overall GlcN production (Álvarez-Añorve et al., 2005; Chen et al., 2012). Besides, *E. coli* can use GlcN and GlcNAc as alternative carbon sources (Álvarez-Añorve et al., 2005; Deng et al., 2005). Intracellular GlcNAc-6-phosphate can be deacetylated by GlcN-6-phosphate deacetylase (*nagA*), resulting in GlcN-6-phosphate, which undergoes deamination by GlcN-6-phosphate deaminase (*nagB*), producing fructose-6-phosphate, thereby, leading to subsequent degradation via glycolytic pathway. GlcNAc-6-phosphate is an allosteric activator of GlcN-6-phosphate deaminase. These catabolic enzymes are organized in a cluster (*nagBACD* operon), where nagC acts as a regulator in the GlcN and GlcNAc degradative pathway (Álvarez-Añorve et al., 2005; Deng et al., 2005; Liu et al., 2013). To prevent product loss, this catabolic pathway gene cluster, *nagBACD*, should be deleted. Moreover, a mannose transporter (*manXYZ*) and a GlcNAc-specific uptake transporter (*nagE*) are used for transporting and phosphorylating GlcN and GlcNAc inside the cell, respectively (Liu et al., 2013), which are then further utilized as carbon and nitrogen sources, lowering the yield of the end products (Deng et al., 2005; Liu et al., 2013). The toxicity of intracellular GlcN-6-P can be resolved by cloning GlcN-6-phosphate N-acetyltransferase gene (*gna1*), which adds up one more step in the GlcN production pathway, diverting GlcN into GlcNAc, a relatively stable and less toxic end-product (Deng et al., 2005).

**Fig. 6.**
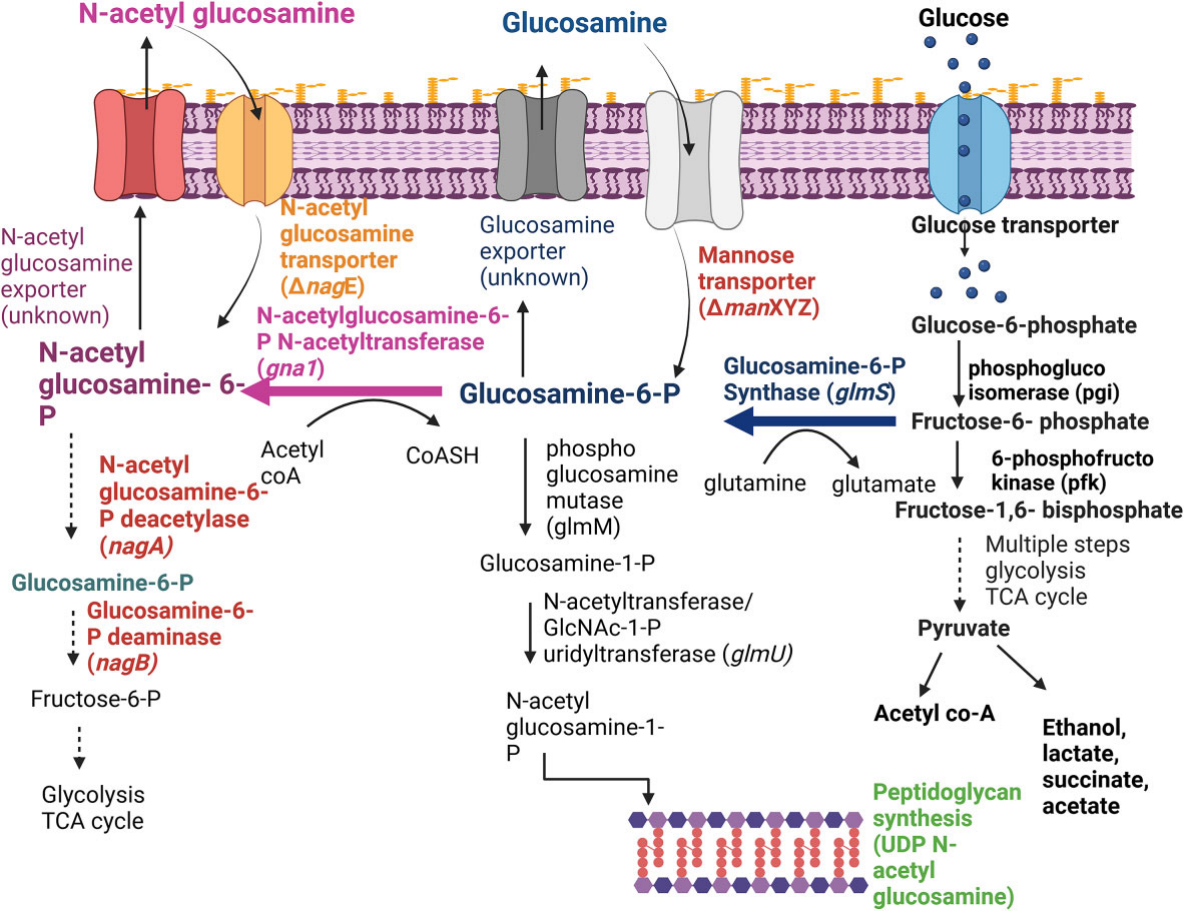
Overview of GlcN and GlcNAc production and degradation pathway, and peptidoglycan synthesis pathway in *E. coli* (https://BioRender.com/k72b864).

Metabolic flux balance is a crucial factor in controlling the GlcN/GlcNAc biosynthetic pathway over cell growth. The competition for carbon resources between cell growth and product synthesis is a significant challenge in the efficient production of GlcNAc in microbial cell factories. Because the production of GlcNAc from fructose-6-P directly competes with central carbon metabolism, the amount of glucose needed for cellular metabolism and cell growth significantly restricts the amount of GlcNAc (Ma et al., 2021). Overall, there are three primary strategies for GlcN and GlcNAc synthesis using recombinant bacteria, (1) Overexpression of key enzyme for GlcN and GlcNAc synthesis; (2) Deletion of GlcN and GlcNAc uptake transporter and catabolic pathway genes; and (3) Deletion of other byproduct pathway genes to divert more carbon flux into the GlcN and GlcNAc production pathway. The overall pathway for GlcN and GlcNAc production using metabolic engineering of bacteria is illustrated in Fig. 6.

An *E. coli* strain was developed by Deng et al. for the production of GlcN. This strain was initially engineered to overexpress the *glmS* gene using the*T7lac*-expression cassette system. The GlcN transporter gene (*man*XYZ operon) and the GlcNAc catabolic genes (*nag* regulon) were then deleted using P1 phage transduction. During fermentation, lactose was used to replace the expensive IPTG to induce *T7lac-controlled glmS* expression. By switching between lactose and glucose feeding, this engineered strain produced 17 g/L of GlcN, achieving limited success. The presence of catabolite repression and GlcN toxicity (40 g/L of GlcN results in complete inhibition of cell growth) accounted for this undesirable product titer. Overexpression of the GlcN-6-phosphate N-acetyltransferase gene (*GNA1*) from *Saccharomyces cerevisiae* converted GlcN-6-P into GlcNAc. Under improved conditions, the strain produced 110 g/L of GlcNAc in fed-batch fermentation (1 L fermenter, pH 6.9, 37°C, 72 hr) upon induction by IPTG, with a product yield of 82% by weight (Deng et al., 2005). Using a similar approach, another genetically engineered *E. coli* strain (*E. coli-glmS-gna1*) was developed by Chen et al. (2012). By improving a stepwise dissolved oxygen control mechanism, GlcN and GlcNAc increased to 72.89 g/L using IPTG as the inducer (Chen et al., 2012). The fermentation parameters are 3 L fermenter, pH 7.0, 37°C, DO 20%–40%, 24 hr. However, the *T7lac-*expression cassette system had certain limitations for large-scale applications. The fermentation substrate (glucose) affected the lac promoter's positive regulation due to catabolite repression (glucose effect). The host strain engineered in both of the studies had competing fermentation pathways, which led to the accumulation of the side products such as lactate, acetate, succinate, and ethanol into the fermentation medium, which decreased the product yield and inhibited host cell growth (Chen et al., 2012; Deng et al., 2005).

Advances in synthetic biology, systems biology, and control theory have provided the tools and design principles necessary for dynamic metabolic engineering, and there are several examples where dynamic metabolic control has enabled microbes to produce higher quantities of metabolite products (Hartline et al., 2021). Zhang et al. introduced a T7 RNA polymerase-PT7 promoter system induced by xylose to control GlcNAc synthesis, along with an arabinose-induced CRISPR interference (CRISPRi) system to regulate cell growth by decreasing the transcription of key growth-related genes, *pfkA* and *zwf*, encoding for 6-phosphfructokinase and glucose-6-phosphate 1-dehydrogenase, respectively. Optimization of the timing and concentration of the two inducers, the carbon flux between cell growth and GlcNAc synthesis was redistributed in a way that reduced the TCA cycle and allowed more carbon to be used for GlcNAc synthesis, which produced 90.9 g/L GlcNAc in 40 hr (Zhang et al., 2020). Here, the fermentation conditions are: fed batch, 5 L, pH 7.0, 37°C, DO 20%–40%, 48 hr.

Ma et al. reported an improved *E. coli* strain with *pfkA* gene deletion and mutation of *glpK* gene (encoding glycerol kinase) to promote glycerol utilization to support cellular metabolism and to save maximum glucose for GlcNAc production pathway. The improved strain had a yield of 179.7 g/L GlcNAc using mixed glycerol/glucose (1:8, m/m) carbon sources in a 5 L bioreactor (pH 7.0, 37°C, DO 20%–30%, 60 hr) (Ma et al., 2021). A similar glycerol catabolic approach was used in another article to maintain cell viability and produce amino and acetyl groups of GlcNAc in *E. coli*. Glycolysis was interfered by deleting *pfkA, pfkB*, and *zwf* genes, co-utilization was improved by overexpression of *glpK* gene and GlcNAc production was developed by overexpression of *glmS, GNA1*, and *YqaB* gene (encoding GlcNAc-6-phosphate phosphatase). The resulting strain co-utilized glucose and glycerol had a 0.64 mol yield of GlcNAc per mol of glucose consumed (Wang et al., 2022). One study used genome-scale metabolic network model to analyze flux balance for GlcNAc overproduction using an engineered *E. coli*. The strain improvement steps include introduction of the GlcNAc synthetic genes into the host, knocking out of *poxB* (pyruvate dehydrogenase), *ldhA* (lactate dehydrogenase), *murQ* (N-acetylmuramic acid 6-phosphate etherase) and *glnA* (glutamine synthetase) genes, and CRISPRi based interference of *pfkA* and *zwf* genes for balancing carbon flux. The final strain had a 143.8 g/L GlcNAc overproduction in a 30-L bioreactor (Lu et al., 2022). The production of glucosamine using different *E. coli* strain is summarized in Table 1.

**Table 1.** List of recombinant *E. coli* strain for GlcN and GlcNAc production

Host used	Genes inactivated/deleted	Genes overexpressed	Titer of GlcN and/or GlcNAc	Remarks	Fermentation conditions	References
*E. coli* (DE3)	*ΔmanXYZ, ΔNagBACD, ΔnagE*	*glmS* and *GNA1*	17 g/L GlcN, 110 g/L GlcNAc,	Introducing *GNA1* to the GlcN pathway overcomes the toxic effect of GlcN on host cell	Fed batch, 1 L fermenter, pH 6.9, 37°C, 72 hours.	Deng et al. (2005)
*E. coli* (DE3)		*glmS* and *GNA1*	72.89 g/L GlcNAc	Stepwise dissolve oxygen (DO) control increased the yield of GlcN and GlcNAc production	3 L fermenter, pH 7.0, 37°C, DO 20%–40%, 24 hr	Chen et al. (2012)
*E. coli* W3110	*ΔnagE*, Interference of *pfkA* and *zwf* by CRISPRi	*glmS* and *GNA1*	90.9 g/L GlcNAc	*pfkA* and *zwf* repression directed carbon flux towards the GlcNAc synthesis	Fed batch, 5 L, pH 7.0, 37°C, DO 20%–40%, 48 hr	Zhang et al. (2020)
*E. coli* W3110	*ΔnagBAC, ΔmanXYZ, ΔpfkA*	*glmS, GNA1* and *glpK*	179.7 g/L GlcNAc	Glycerol is utilized for cell survival, saving more glucose into GlcNAc production	Fed batch, 5 L, pH 7.0, 37°C, DO 20%–30%, 60 hr	Ma et al. (2021)
*E. coli*	*Δ*PfkA, *Δ*PfkB, *Δ*zwf	*glmS, GNA1, glpK*, and *YqaB*	0.64 mol/mol	Glucose/glycerol co-utilization used to maintain cell viability and produce amino and acetyl groups	Batch, 1 L, 37°C, 36 hr	Wang et al. (2022)
*E. coli* (DE3)	*Δ*manXYZ, *Δ*nagABE, *Δ*FucIK *ΔpoxB, ΔldhA, ΔmurQ*, CRISPRi based interference of *pfkA* and *zwf*	glmS, GNA1	143.8 g/L of GlcNAc	Metabolic engineering strategies were generated through genome-scale metabolic network analysis	Fed batch, 30 L, pH 7.0, 37°C, DO 30%–50%, 55–65 hr	Lu et al. (2022)

### Optimization of Fermentation Conditions and Downstream Processing

Carbon sources are an essential factor in GlcN and GlcNAc production. Glucose is the preferred carbon source, as it is readily available and is efficiently metabolized by most microorganisms (Deng et al., 2005). As these amino sugars require glutamine as an amino group donor, the media should supplement a significant amount of ammonium salts. Several studies designed the production media using different concentrations of salts, trace minerals, and a buffering agent (Deng et al., 2005; Lu et al., 2022). The induction time, pH, temperature, and agitation rate also play an important role in production. According to Deng et al., GlcN was degraded at neutral pH, and the late-stage pH of the fermentation medium was shifted to pH 5.0 for better GlcN production (Deng et al., 2005). Both GlcN and GlcNAc can inhibit cell growth. Therefore, a two-step metabolic control model is necessary. The initial phase of the process involves engineering cells for rapid growth without production. In the subsequent phase, the growth of the cells is restricted while carbon flux is directed toward the production of the desired product (Hartline et al., 2021). Often the formation of mixed acids will lower the pH, hindering the optimum cellular growth (Deng et al., 2005). After fermentation, the GlcN and GlcNAc are extracted and purified depending on the host strain type (bacteria or fungi), secretion system (intracellular or extracellular), and fermentation methods. In bacterial cells, GlcN and GlcNAc are released outside the cell using specific transporters, which makes the cell-media separation process less complicated than fungal biomass separation (Liu et al., 2013). After the fermentation is complete, the media is centrifuged to separate the cell pellet from the supernatant. The supernatant is then collected, decolorized, and concentrated in a vacuum chamber to obtain a saturated solution containing GlcN and/or GlcNAc, and other byproducts, such as mixed acids. The resulting mixture is then purified using ethanol precipitation and low-temperature crystallization, which increases the GlcN and GlcNAc concentration while separating from impurities (Ahuja et al., 2021). GlcN is generated from GlcNAc from mild acid hydrolysis (Sitanggang et al., 2012). For fungal fermentation, acid hydrolysis with strong hydrochloric acid and high temperature is used to obtain semi-pure GlcN and GlcNAc from solid biomass (Hsieh et al., 2007). The GlcN and GlcNAc can be qualitatively analyzed using Thin Layer Chromatography (Lv et al., 2017). It can be quantified using the colorimetric method (Elson & Morgan, 1933; Roseman & Daffner, 1956). The High-Performance Liquid Chromatography technique (Kraisangsri et al., 2018) coupled with Mass Spectroscopy might be used for further purification, characterization, and quantification used (Kumar et al., 2020). However, GlcN and GlcNAc are polar and water-soluble molecules, making their separation from other polar compounds in a biological matrix difficult. The overall low concentration of these amino sugars in biological samples, their chemical similarity to other compounds, and the complex media components such as salts, proteins, and lipids can cause ion suppression or enhancement, leading to inaccurate quantification (Kim et al., 2015). Therefore, developing an efficient HPLC method for detecting glucosamine in a biological matrix requires careful optimization of sample preparation, chromatographic conditions, and detection parameters to overcome these challenges.

## Conclusion

Microbial approaches have shown great promise in the production of GlcN and GlcNAc. Overall, microbial production of GlcN and GlcNAc has the potential to provide sustainable and cost-effective sources of these compounds for various industrial applications, including pharmaceuticals, nutraceuticals, and cosmetics. So far, metabolic engineering techniques have been successfully applied to redirect metabolic pathways and enhance the production of these compounds in various microorganisms. Although more aligned with the principles of green chemistry by using low-cost raw materials and mild reaction conditions, microbial GlcN and GlcNAc production methods still have challenges that need to be addressed, such as the minimization of byproduct formation, effective distribution of metabolic flux towards production process, optimization of fermentation conditions and the development of efficient downstream processes.

## Data Availability

No new data was generated or analyzed in this research.
